# Supplementary material for: Hybrid supercapacitors for reversible control of magnetism
Source: Nat Commun. 2017 May 10;8:15339. doi: 10.1038/ncomms15339 (PMC5436217; doi:10.1038/ncomms15339)
Supplement: Supplementary Information — Supplementary Figures, Supplementary Tables, Supplementary Notes and Supplementary References. [file ncomms15339-s1.pdf]

SUPPLEMENTARY NOTE 1. SYNTHESIS AND CHARACTERIZATION OF LSMO THIN FILMS

LSMO thin films were grown by on-axis rf-sputtering in a custom-made deposition chamber (Createc Fischer GmbH). The unconventionally large target-sample separation of ≈285 mm allowed for the fabrication of smooth and homogeneous films over surface areas of about 0.4 cm^2 on top of single-crystal $SrTiO_3$ (STO) substrates. The chamber base pressure was in the order of $\sim 10^{-9}$ mbar. The LSMO films were prepared using a deposition temperature of ≈650 °C at a pressure of 0.018 mbar in a argon-oxygen mixture (with a 3/2 ratio) and a growth rate of $\approx 0.4\,\text{Å}\,\text{s}^{-1}$. At the end of the deposition the samples were cooled down to room temperature at a rate of 10 K min^{-1} in a pure oxygen atmosphere (0.08 mbar). Comparing to the deposition conditions reported elsewhere[1], where more details about the growth conditions can be found, a post annealing step was performed in air at 900 °C for one hour in order to reduce the amount of oxygen deficiencies. This process brought a significant increase in T_C from ∼270 K to ∼320 K without negatively affecting the film morphology (see below). Some deposition tests were also performed on chemically-treated STO substrates[2], but no remarkable improvements in the final films properties were found.

Before performing *in situ* tuning measurements of magnetic response upon surface charging/discharging, the LSMO samples were thoroughly characterized. As shown in Fig. 6b,c, tapping-mode atomic force microscopy (AFM) revealed a smooth surface morphology of the LSMO films. The crystal structure and the interface between substrate and film were studied via scanning transmission electron microscopy (STEM). Perfect fully-strained cube-on-cube growth of LSMO on the top of STO was found (Supplementary Figure 1a). X-ray reflectivity measurements (not shown) revealed the presence of sharp interfaces and a film thickness of ≈13 nm. High-resolution X-ray diffraction (HRXRD) confirmed the expected $< 001 >$-oriented growth and exhibited pronounced Laue oscillations (Supplementary Figure 1b), whose presence indicate high-quality epitaxial growth. The measured out of plane lattice parameter of LSMO was ≈3.86 Å. The full width at half maximum (FWHM) of the rocking curves of the (001) and (002) reflections (not shown) was around 0.06°, which is comparable to the values reported in literature for LSMO thin films prepared with pulsed laser deposition[3] and molecular beam epitaxy[4].

The chemical composition was analyzed by Rutherford backscattering spectroscopy (RBS) for a sample grown on MgO substrate following the same deposition parameters used with STO in order to avoid the overlap of the strontium signals (Supplementary Figure 1c). Quantitative analysis with the Mn stoichiometry normalized to 1 revealed a strontium content of 0.26 within an accuracy of 2%. The magnetic properties were characterized by Superconducting Quantum Interference Device (SQUID) magnetometry (Supplementary Figure 1d). The analysis of the derivative of the field-cooled measurements performed in the 2 - 400 K temperature range with an external magnetic field of 100 Oe parallel to the plane of the film resulted in a Curie temperature of $T_C \approx$ 323 K and a markedly sharp magnetic transition (FWHM of 14 K). The increase in magnetization at around 105 K is attributed to the magnetostriction in LSMO induced by the structural phase transition of STO from cubic to tetragonal, which underlines once again the excellent substrate-film adhesion. A total saturation magnetization value of $M_{sat} \approx 2.7\,\mu_B\,\text{u.c.}^{-1}$ was determined by a magnetic hysteresis loop measurement using a maximum magnetic field of 10 kOe at a temperature of 130 K (see inset in Supplementary Figure 1d). The theoretical bulk value[5] of $M_{sat} = 3.74\,\mu_B\,\text{u.c.}^{-1}$, usually not reachable in thin films, can be obtained under the assumption of a magnetically dead layer of about 3.5 nm at the LSMO/STO interface, a rather reasonable value comparing to other literature reports[6].

SUPPLEMENTARY NOTE 2. TEMPERATURE DEPENDENCE TUNING OF MAGNETISM UPON CONSTANT ΔQ

During the *in situ* tuning experiment carried out in the 220–330 K temperature range with a constant surface charge modulation $\Delta Q \approx 4\,\mu\text{C}\,\text{cm}^{-2}$, the current density-voltage characteristics (see Supplementary Figure 2) displayed nearly symmetric and rectangular shapes of the charging and discharging processes as well as high reversibility upon cycling. The behavior of the magnetic response as a function of temperature is shown in Supplementary Figure 3. The slope of the curves, used to calculate the magnetoelectric (ME) coupling coefficients, revealed positive or negative values of α respectively above or below $T_{cross} \approx 258$ K.

Interestingly, high and positive values of $\alpha \approx +6.4\,\mu_B/h^+$, which exceed the magnetic moment carried by a Mn atom, were found in proximity of the para-ferromagnetic phase transition. The behavior might be ascribed to the enhanced penetration depth λ of the local electric field on insulating domains, which according to phase separation models[7,8] are present in larger amounts comparing to metallic domains around T_C. This interpretation is corroborated by the fact that upon decreasing the temperature also the magnitude of α systematically decreases, which is consistent with the expected increased number of conductive domains and consequent reduction of λ (on average on the whole LSMO surface).

At temperatures below T_{cross}, starting from ≈ 235 K down to 220 K, α reaches a nearly constant value of $\approx -3\,\mu_B/h^+$, in agreement with the fact that the LSMO magnetization approaches saturation (and so also its magnetoelectronic

configuration does not remarkably change).

In Supplementary Table 1 (see below) the values of potential window, measured charge, calculated capacitance and ME coupling coefficient obtained from the *in situ* temperature dependence survey are summarized.

SUPPLEMENTARY NOTE 3. ADDITIONAL INSIGHTS INTO THE QUALITATIVE EXPLANATION OF THE INTERFACIAL ME PHENOMENA

As already stated in the main paper, a phenomenological model based on the LSMO bulk phase diagram can be used to qualitatively describe the $La_{0.74}Sr_{0.26}MnO_3$ magnetic response upon surface charge doping. The competition between the change in magnetic transition temperature and ground state magnetization upon charge carrier doping has already been used[9–12] to explain the existence of a crossover point T_{cross}. Naively this can be illustrated by a shift of T_{C} to higher temperatures and, concurrently, to a reduced saturation magnetization when the system is upon hole doping, and viceversa upon electron doping (see Supplementary Figure 4).

To our knowledge, for the first time the results of the isothermal-charge dependence study at 220 K (see Fig. 4) prove that it is possible to control at a fixed temperature the sign of the magnetic modulation by simply adjusting the external voltage analogously to the behavior observed below or above T_{cross} in the temperature dependence study (see Fig. 3).

The phenomenological picture, notwithstanding its limitations, is still helpful to qualitatively interpret the results at 220 K upon progressive increasing ΔQ. Using as a starting point the profile of the bulk, untuned $M(T)$ curve (black curve in Supplementary Figure 4), progressive electron doping can be described with a gradual shift of T_{C} towards higher temperatures simultaneously accompanied by a decrease in ground state magnetization (red curves in Supplementary Figure 4). The opposite trend is expected upon hole doping (blue curves in Supplementary Figure 4).

The insets in Supplementary Figure 4 represent the behavior expected below and above T_{cross}, labeled with the arbitrarily-chosen temperatures of 220 K and 270 K, respectively.

Considering the inset below T_{cross} in Supplementary Figure 4, on the one hand an initial increase in magnetization up to a maximum and afterwards a decrease in its value are expected upon progressive electron doping (see the red symbols at 220 K). On the other hand, upon hole doping, the consequent increase in T_{C}, which is not playing the major role at the low temperature of 220 K, and decrease in the ground state magnetization, bring to a systematic decrease in magnetization (blue symbols at 220 K). These behaviors are consistent with the trend of the isothermal charge-dependence study performed at 220 K (see Fig. 4).

Similar considerations are also valid to describe the inset above T_{cross}. However, in this case a reversed effect is expected upon charge doping: an initial increase and afterwards a decrease in magnetization upon increased *hole* concentration (red symbols at 270 K), while a systematic decrease in magnetization upon electron doping (blue symbols at 270 K). In this sense, contrary to the results obtained at 220 K, a splitting of the $M(Q)$ curve at temperatures above T_{cross} should be manifested upon progressive *hole* accumulation. This prediction, despite based on a relatively simple and qualitative model, is confirmed by the results of an isothermal charge-dependence study performed at 270 K (see Supplementary Note 4).

Furthermore, it should be stressed that this qualitative scenario, based on the shift of the bulk, untuned $M(T)$ curve, can be used only as a first rough approximation. Additional parameters, such as magnetoelectronic phase separation with possible variations in the electric field penetration depth and the quantitative values of α, should be considered to build a more realistic model.

A last speculative remark pertains to the nearly symmetric shape of plot e in Fig. 4, characterized by positive or negative slopes (i.e. α) at the fixed temperature of 220 K. It is known from the temperature dependence study that T_{cross} is $\approx 258\,\text{K}$ and it separates the regions with positive or negative α. Thus, one could argue that with respect to the two opposite extremes of the hole accumulation/depletion branches (see plot e in Fig. 4), the LSMO surface magnetic configuration shifts ~160 K in total. Analogously, the splitting in the $M(t)$ curve at 323 K upon hole doping (see Fig. 6a), suggests a total shift of ~65 K. The authors underline that these hypotheses require to be verified by independent experiments and an adequate theoretical model.

SUPPLEMENTARY NOTE 4. ISOTHERMAL CHARGE-DEPENDENCE STUDY AT 270 K

In analogy to the isothermal charge-dependence experiment at 220 K, the LSMO magnetic response was analyzed upon progressive expansion of the potential window at a fixed temperature of 270 K, i.e., above T_{cross}.

For potential windows below ~0.8 V, the magnetic signal was in-phase with respect to the surface charge modulation (a in Supplementary Figure 5a), in agreement with the temperature-dependence study (see Fig. 3). Beyond this threshold voltage, the $M(t)$ curve manifested a splitting on the hole accumulation side (b in Supplementary Figure 5a)

with the distinctive anti-phase characteristic with respect to $Q(t)$, as observed *below* T_{cross}. As the potential window was further increased (c,d in Supplementary Figure 5a), this behavior became more and more pronounced. Upon application of a positive bias $V_{\mathrm{b}} \approx +0.9\,\mathrm{V}$, the magnetic signal turned out to be directly anti-phase with the interface charge modulation (e in Supplementary Figure 5a). It should be noticed the similarity with the results obtained at 220 K, keeping in mind the reversed role of the hole/electron doping in controlling the magnetic response, which supports the considerations discussed in Supplementary Note 3.

The trend of Supplementary Figure 5b, featuring positive or negative values of α respectively along the hole depletion or accumulation branches, suggests once again that upon increasing ΔQ the system behaves as being toggled back and forth with respect to T_{cross}.

The shape and reversibility of the $J(V)$ characteristics (see Supplementary Figure 5c) and the values of calculated capacitance confirm that upon expansion of ΔV, the charging/discharging processes in the LSMO/ionic liquid system are due to a combination of electric-double-layer capacitance and surface pseudocapacitance. The values of potential window, surface charge, capacitance and ME coupling coefficient are summarized in Supplementary Table 3.

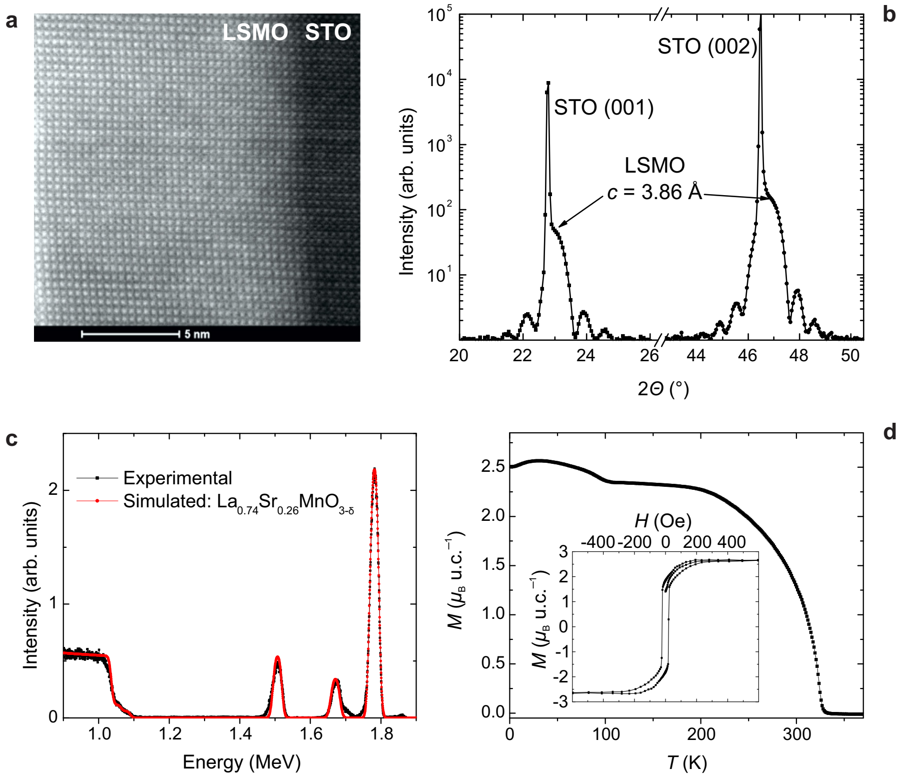

Supplementary Figure 1 | LSMO films characterization. **a**, STEM micrograph of LSMO grown on STO substrate. **b**, HRXRD θ–2θ diffractogram in proximity of the (001) and (002) STO substrate reflections. **c**, Rutherford backscattering spectroscopy spectrum of a $La_{0.74}Sr_{0.26}MnO_3$ film grown on MgO substrate. **d**, Field-cooled magnetization measurement performed in the 2 - 400 K temperature range. The inset in **d** shows a magnetic hysteresis loop measurement performed at 130 K.

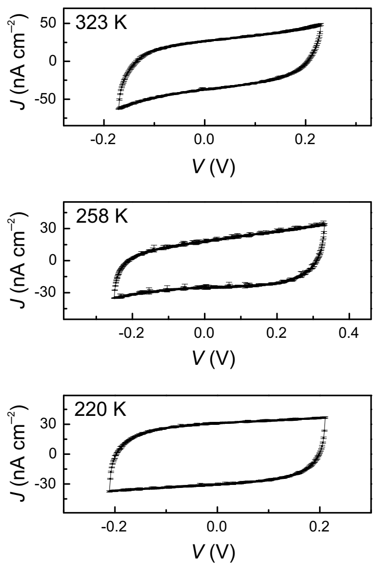

Supplementary Figure 2 | Current-voltage characteristics at 323 K, 258 K and 220 K. The curves feature a high level of reversibility and rectangular-like shapes, as expected for a traditional electric-double-layer capacitor. The curves are constructed by averaging several consecutive cycles (40 for the measurements at 323 and 220 K and 10 for the plot at 258 K). The three plots belong to the same set of data already shown in Fig. 3d-f, respectively. Where not visible, the statistical errors are smaller than the symbols size.

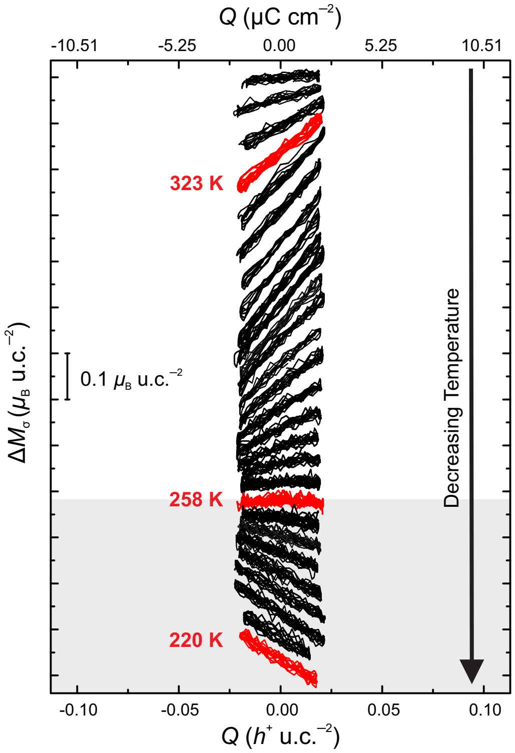

Supplementary Figure 3 | Temperature-dependence magnetic response upon application of a nearly constant surface charge modulation $\Delta Q \approx 4\,\mu\mathrm{C\,cm}^{-2}$. The slope of the curves provides the magnetoelectric coupling coefficient α (see Supplementary Table 1). Each curve consists of at least 10 consecutive CV cycles. Above (white area) or below (gray area) $T_{\mathrm{cross}} \approx 258$ K the tuning coefficient is positive or negative, respectively. The curves at 323 K, 258 K and 220 K belong to the same set of data already shown in Fig. 3d-f, respectively.

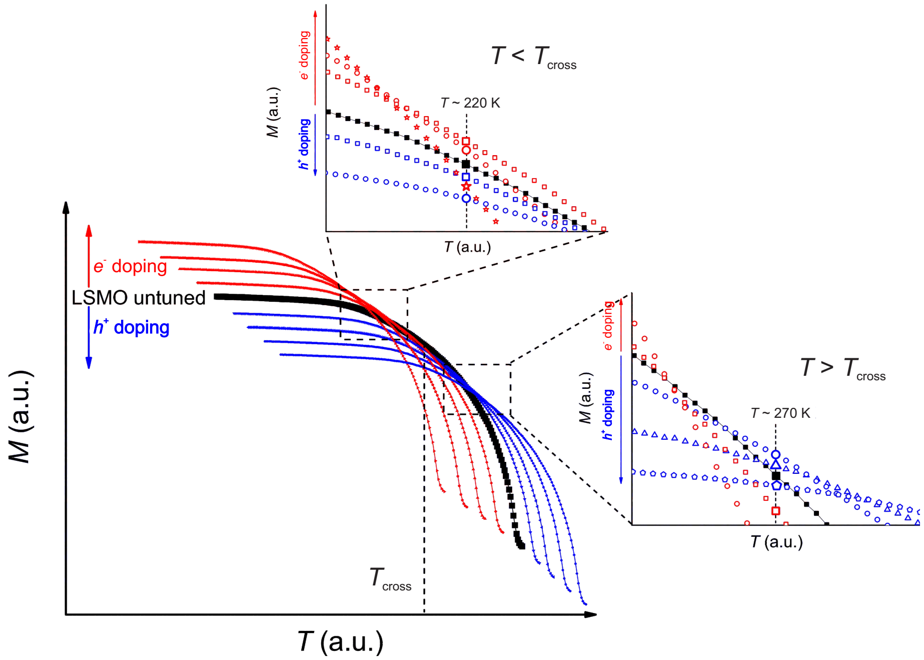

Supplementary Figure 4 | Naive sketch representing the shift of the magnetic FC curves upon progressive electron and hole doping. The insets show the behavior expected at 220 and 270 K, respectively below and above T_{cross}.

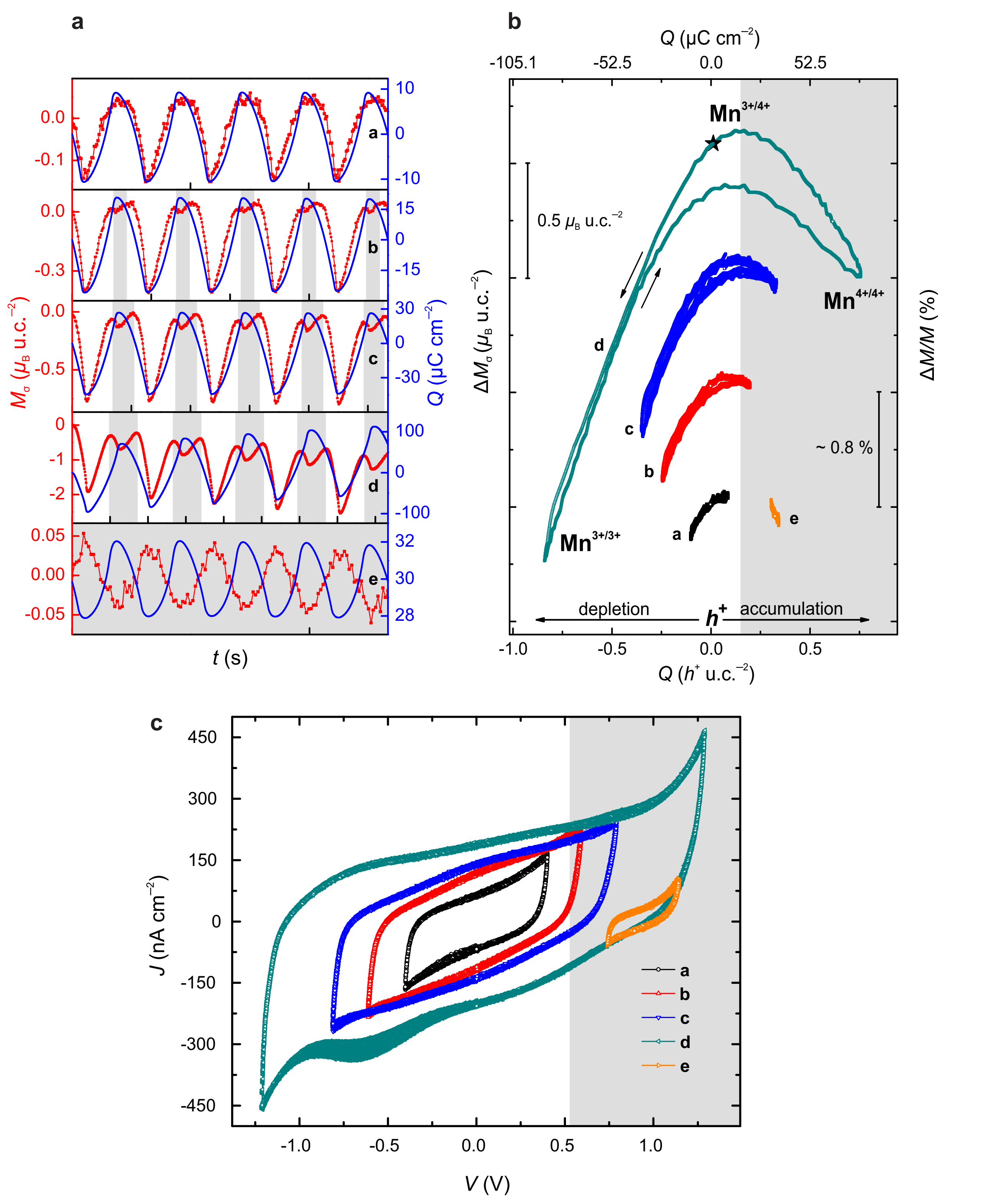

Supplementary Figure 5 | Isothermal charge-dependence study at 270 K. a, Time-resolved magnetic response upon sequential increase of the surface charge modulation at 270 K. Each time step on the abscissa corresponds to 1000 seconds. **b**, Behavior of magnetization as a function of the change in surface doping level. **c**, Current density - voltage characteristics upon expansion of the potential window ($0.8\,\mathrm{V} < \Delta V < 2.5\,\mathrm{V}$). Each curve corresponds to five consecutive CV cycles. Plot "e" in **a**,**b**,**c** refers to an experiment performed with a starting bias voltage $V_\mathrm{b} \approx +0.8\,\mathrm{V}$. In **a**,**b**,**c** the white and gray areas qualitatively separate the data with the LSMO magnetic modulation responding as above or below T_cross, respectively.

Supplementary Table 1 | Summary of the temperature-dependence data: temperature, potential window, surface charge, capacitance and magnetoelectric coupling coefficient. The reported data are within a 5% accuracy.

T (K)	ΔV (mV)	ΔQ (μC cm^{-2})	C (μF cm^{-2})	α (μ_B/h^+)
326	350	3.4	10.4	+0.5
325	380	3.9	10.8	+1.4
324	380	3.6	10.1	+2.7
323	400	3.8	10.1	+4.3
322	420	3.9	9.9	+5.2
321	420	3.7	9.6	+5.5
320	420	3.7	9.4	+5.7
315	440	3.7	8.8	+6.2
310	470	3.8	8.7	+6.4
305	500	4.0	8.5	+6.2
300	500	3.9	8.2	+5.9
295	530	4.0	8.0	+5.3
290	530	3.9	7.8	+4.7
285	550	4.0	7.9	+4.1
280	560	4.1	7.8	+3.2
275	560	4.0	7.8	+2.5
270	560	3.9	7.6	+1.8
265	560	3.8	7.5	+1.0
260	580	3.9	7.4	+0.2
258	580	4.0	7.0	+0.02
255	580	3.7	7.0	-0.5
250	600	3.9	7.0	-1.3
245	600	3.9	6.8	-1.9
240	600	4.1	7.2	-2.6
235	600	4.5	8.0	-2.9
230	550	4.5	8.5	-3.2
225	400	3.3	8.8	-2.9
220	420	3.8	9.7	-3.0

Supplementary Table 2 | Summary of the isothermal charge-dependence study at 220 K: potential window, surface charge, capacitance and magnetoelectric coupling coefficient. The lettering corresponds to the plots in Fig. 4. The data of plot "f" refer to an experiment performed with a starting bias voltage $V_b \approx -1.3$ V. Unless specified, the errors on the reported data are within a 5% accuracy.

Plot	ΔV (mV)	ΔQ (μC cm^{-2})	C (μF cm^{-2})	α (μ_B/h^+)
	200	1.5	8.1	-2.9
	300	2.5	9.0	-3.1
a	400	3.7	9.8	-3.1
	500	5.0	10.6	-2.9
	600	6.5	11.3	-3.0
	700	8.0	12.0	-2.9
	800	9.7	12.6	-2.8
	1000	13.1	13.7	-2.7
	1500	23.3	16.3	-2.6
b	2000	38.0	20.0	-2.5
	2500	57.2	24.3	-2.1
c	2900	87.1	32.1	-1.8(2), +1.3
d	3300	163.4	53.8	-1.7(2), +1.8(2)
e	3700	266.5	85.6	-1.4(6), +2.0(5)
f	300	6.7	27.6	+2.2

Supplementary Table 3 | Summary of the isothermal charge-dependence study at 270 K: potential window, surface charge, capacitance and magnetoelectric coupling coefficient. The lettering corresponds to the plots in Supplementary Figure 5. The data of plot "e" refer to an experiment performed with a starting bias voltage $V_\mathrm{b} \approx +0.8\,\mathrm{V}$. Unless specified, the errors on the reported data are within a 5% accuracy.

Plot	ΔV (mV)	ΔQ (μC cm^{-2})	C (μF cm^{-2})	α (μ_B/h^+)
a	800	19	30	+1.6
b	1200	47	48	+1.8, –0.7
c	1600	72	54	+2.4, –0.6
d	2500	169	83	+2.5, –1.1(3)
e	400	4	18	–2.0

SUPPLEMENTARY REFERENCES

1. Leufke, P. M. *et al.* Large-distance rf- and dc-sputtering of epitaxial $La_{1-x}Sr_xMnO_3$ thin films. *Thin Solid Films* **520**, 5521–5527 (2012).
2. Kareev, M. *et al.* Atomic control and characterization of surface defect states of TiO_2 terminated $SrTiO_3$ single crystals. *Appl. Phys. Lett.* **93**, 061909 (2008).
3. Aruta, C., Balestrino, G., Tebano, A., Ghiringhelli, G., & Brookes, N.B. Cooperative enhancement of in-plane orbital ordering by oxygen deficiency and in-plane tensile strain in $La_{0.7}Sr_{0.3}MnO_3$ thin films. *Europhys. Lett.* **80**, 37003 (2007).
4. Santos, T., May, S., Robertson, J., & Bhattacharya, A. Tuning between the metallic antiferromagnetic and ferromagnetic phases of $La_{1-x}Sr_xMnO_3$ near x=0.5 by digital synthesis. *Phys. Rev. B* **80**, 155114 (2009).
5. Xiong, X., *et al.* Correlation between coherent Jahn-Teller distortion and magnetic spin orientation in $La_{1-x}Sr_xMnO_3$. *Phys. Rev. B* **60**, 10186-10192 (1999).
6. Angeloni, M., *et al.* Suppression of the metal-insulator transition temperature in thin $La_{0.7}Sr_{0.3}MnO_3$ films. *J. Appl. Phys.* **96**, 6387–6392 (2004).
7. Moreo, A., Yunoki, S. & Dagotto, E. Phase Separation Scenario for Manganese Oxides and Related Materials. *Science* **283**, 2034–2040 (1999).
8. Becker, T. *et al.* Intrinsic Inhomogeneities in Manganite Thin Films Investigated with Scanning Tunneling Spectroscopy. *Phys. Rev. Lett.* **89**, 237203 (2002).
9. Molegraaf, H. J. A. *et al.* Magnetoelectric effects in complex oxides with competing ground states. *Adv. Mater.* **21**, 3470–3474 (2009).
10. Leufke, P. M., Kruk, R., Brand, R. A. & Hahn, H. In situ magnetometry studies of magnetoelectric LSMO/PZT heterostructures. *Phys. Rev. B* **87**, 094416 (2013).
11. Mishra, A. K., Darbandi, A. J., Leufke, P. M., Kruk, R. & Hahn, H. Room temperature reversible tuning of magnetism of electrolyte-gated $La_{0.75}Sr_{0.25}MnO_3$ nanoparticles. *J. Appl. Phys.* **113**, 033913 (2013).
12. Vaz, C. A. F. *et al.* Temperature dependence of the magnetoelectric effect in $Pb(Zr_{0.2}Ti_{0.8})O_3/La_{0.8}Sr_{0.2}MnO_3$ multiferroic heterostructures. *Appl. Phys. Lett.* **97**, 042506 (2010).
